# A type of pancreatic cancer cells form cell clusters from a solitary condition in a primary ciliogenesis-dependent manner

**DOI:** 10.1007/s00795-025-00428-0

**Published:** 2025-03-12

**Authors:** Kenji Shirakawa, Ryota Nakazato, Tetsuhiro Hara, Kenichiro Uemura, Faryal Ijaz, Shinya Takahashi, Koji Ikegami

**Affiliations:** 1 Department of Surgery, Graduate School of Biomedical and Health Sciences, Hiroshima University, 1-2-3 Kasumi, Minami-ku, Hiroshima, 734-8553 Japan; 2 Department of Anatomy and Developmental Biology, Graduate School of Biomedical and Health Sciences, Hiroshima University, 1-2-3 Kasumi, Minami-ku, Hiroshima, 734-8553 Japan

**Keywords:** Pancreatic cancer, Pancreatic ductal adenocarcinoma, Primary cilia, Ciliogenesis, Solitary condition, Cell cluster

## Abstract

Primary cilia are hair-like projections that protrude on most of mammalian cells and mediate reception of extracellular signals. Numerous studies have demonstrated that a variety of cancer cells including pancreatic ductal adenocarcinoma (PDAC) fail to form primary cilia. The loss of primary cilia is thought to cause carcinogenesis and progressive cell proliferation. However, the relationship of the primary cilia loss with carcinogenesis and/or cancer malignancy remains arguable. We herein examined whether ciliogenesis was increased in a model of more progressive PDAC and investigated effects of ciliogenesis on growth of PDAC using a pancreatic cancer cell line, PANC-1. The majority of PANC-1 cells in a cell cluster grown from a solitary cell possessed primary cilia. The rate of ciliogenesis was higher in cells grown from low density than in cells grown from high density. Almost all clones passing limiting dilution culture had abilities to grow primary cilia. Compared to the parental PANC-1 cells, clones that proliferated from a solitary cell showed increase in the ciliogenesis rate. Blocking ciliogenesis suppressed cell cluster formation. Our results suggest that pancreatic cancer cells that are more resistant to a solitary condition have abilities of ciliogenesis and form tumor-like cell clusters in a primary cilia-dependent manner.

**Supplementary Information:**

The online version contains supplementary material available at 10.1007/s00795-025-00428-0.

## Introduction

Pancreatic ductal adenocarcinoma (PDAC), one of the most lethal human cancers worldwide, is anticipated to become the second-leading cause of cancer-related death by 2030 [1]. In recent years, while the efficacy of neoadjuvant chemotherapy and postoperative adjuvant chemotherapy has also been reported, the survival prognosis has not been significantly improved [2, 3]. This is thought to occur partly due to strong intratumour heterogeneity because PDAC accumulates genetic abnormalities even after tumor formation [4]. In addition to the intratumour heterogeneity, characteristics of PDAC cells able to grow from solitary conditions are thought to be another reason for the poor prognosis of PDAC. To support the idea, micrometastasis with a few PDAC cells, which is difficult to be detected by conventional hematoxylin and eosin staining, is found in para-aortic lymph nodes and identified as a poor prognostic factor for PDAC [5]. Moreover, circulating tumor cells in plasma are another poor prognostic factor for PDAC [6, 7]. Due to these cellular and molecular complexities of this disease, PDAC treatment requires a personalized strategy. New effective biomarkers and poor prognostic factors are thus urgently desired to be found for diagnosis, disease monitoring, and treatment options against PDAC.

Almost all cells, from the embryonic development to adulthood, form a hair-like membranous protrusion called the primary cilium [8–10]. The core structure of primary cilium is comprised of a rotationally symmetric arrangement of 9 doublet microtubules, so-called the axoneme, which extends from a centriole, one of a paired cylinder-like structure in the centrosome. The primary cilia harbor key molecules that mediate signaling pathways important for development, such as the Hedgehog pathway, Wnt pathway, and platelet-derived growth factor pathway [11–14]. Hereditary malformation or dysfunction of primary cilia causes several genetic disorders called ciliopathies, which include Bardet-Biedl syndrome, Joubert syndrome, and polycystic kidney disease [15–17]. An underlying mechanism of ciliopathies involves dysregulation of cell cycle. Primary cilia are assembled in G0 or G1 phase of proliferative cells or in non-dividing cells, while shortened or disassembled upon stimulation by specific growth factors, which trigger the cell cycle to re-drive into G1/S phase from G0 phase [18]. Conversely, primary cilia assembly is also thought to dictate the cell cycle; keeping primary cilia formed arrests cell cycle at G0 phase [19], while the failure of primary cilia formation accelerates cell cycle progression [20].

A large amount of studies have demonstrated that primary cilia defects strongly correlate with cancer progression [21, 22]. However, the relationship of primary cilia with cancers is still controversial in a same carcinoma type as well as among different carcinoma types. Primary cilia are reported to be required for tumorigenesis in medulloblastoma and basal cell carcinoma [23, 24], while they are lost in a vast majority of cancers. PDAC cells were thought to lose primary cilia during transmutation from acinar-to-ductal metaplasia through pancreatic intraepithelial neoplasia [25, 26]. A Japanese group has reported that suppressing primary cilia formation enhance cell proliferation in a cell line of PDAC, PANC-1 [27, 28]. In contradiction to the prior works, the same group has reported that restoration of primary cilia enhances cell proliferation in PANC-1 cells [29]. A clinicoepidemiological study supports the latter finding. Almost all PDAC cases where primary cilia were detected had lymph node metastasis, while those lacking primary cilia had lower lymph node metastasis [30]. Patients with primary cilia observed in PDAC had a poorer prognosis compared with patients without primary cilia [30]. Thus, impacts of primary cilia formation or defects on PDAC progression are still arguable.

In this study, we examine whether primary cilia affect positively or negatively the growth of a type of PDAC by using PANC-1 cells. In particular, since PDAC cells are considered capable of proliferating from solitary conditions and to have high proliferative potential [5–7], we focus on the relationship between primary cilia formation and the ability of cell cluster formation from a solitary cell.

## Materials and methods

### Cell culture

A human pancreatic cancer cell line, PANC-1, was purchased from RIKEN Bioresources Cell Bank (Ibaraki, Japan), and cultured in RPMI-1640 (Wako; Osaka, Japan) supplemented with 10% fetal bovine serum (FBS; Gibco, USA) at 37 ºC in a humidified atmosphere containing 5% CO_2_ as described previously [31]. MIA PaCa-2 (human cell line derived from pancreatic cancer) cells (RIKEN BRC, RCB2094) were cultured in DMEM-low glucose (Wako) with 10% FBS. Cells were harvested from dishes with trypsin, and the number of cells was counted using a hemocytometer. Photographs of cells were acquired using an inverted microscope (AE3000, SHIMADZU RIKA; Tokyo, Japan).

### Antibodies

The following antibodies were used for immunofluorescence staining (IF) and/or western blotting (WB) at each dilutions: ARL13B (1:1000 for IF; rabbit pAb, 17711-1-AP, Proteintech), FOP (1:10000 for IF; mouse mAb, H00011116-M01, Abnova), GAPDH (1:3000 for WB; mouse mAb, MAB374, Millipore), KIF3A (1:1000 for WB; rabbit pAb, 13930-1-AP, Proteintech), IFT88 (1:1500 for WB; rabbit pAb, 13967-1-AP, Proteintech), acetylated α-tubulin (1:10000 for IF; mouse mAb, 6-11B-1, Sigma), γ-tubulin (1:1000 for IF; rabbit pAb, ab11317, Abcam), Alexa fluorophore plus-conjugated secondary antibodies (1:1000 for IF, Thermo), and horseradish peroxidase-conjugated secondary antibodies (1:10000 for WB, Jackson Immuno Research Laboratories).

### Immunocytochemistry

Cells were fixed in 4% paraformaldehyde (PFA, pH 7.5) for 20 min at room temperature. Cells were blocked and permeabilized with 5% normal goat serum containing 0.1% Triton X-100 in PBS (blocking buffer) for 1 h at room temperature. Then, cells were incubated at 4 ºC with primary antibodies diluted in the blocking solution overnight and stained with Alexa Fluor plus-conjugated secondary antibodies and DAPI (1 µg/ml, DOJINDO; Kumamoto, Japan) for 1 h. After washing with PBS, samples were mounted on glass slides with VECTASHIELD mounting medium (Vector Laboratories, Burlingame, CA, USA). Images were acquired using an epifluorescence microscope (DMI3000B, Leica; Tokyo, Japan) and a laser confocal microscope (STELLARIS 5, Leica; Tokyo, Japan).

### Real-time qPCR

Total RNA was extracted from cells and reverse transcribed to cDNA using the PrimeScript 1st Strand cDNA Synthesis Kit (TaKaRa Bio). cDNA was used as a template for real-time qPCR analysis on CFX96 Touch (Bio-Rad) using specific primers targeting glyceraldehyde 3-phosphate dehydrogenase (GAPDH; 5′-GGAGCGAGATCCCTCCAAAAT-3′ as the forward primer and 5′-GGCTGTTGTCATACTTCTCATGG-3′ as the reverse primer), CDH1 (5′-CGAGAGCTACACGTTCACGG-3′ as the forward primer and 5′-GGGTGTCGAGGGAAAAATAGG-3′), CDH2 (5′-TCAGGCGTCTGTAGAGGCTT-3′ as the forward primer and 5′-ATGCACATCCTTCGATAAGACTG-3′ as the reverse primer), VIM (5′-GACGCCATCAACACCGAGTT-3′ as the forward primer and 5′-CTTTGTCGTTGGTTAGCTGGT-3′ as the reverse primer), SNAI1 (5′-TCGGAAGCCTAACTACAGCGA-3′ as the forward primer and 5′-AGATGAGCATTGGCAGCGAG-3′ as the reverse primer), SNAI2 (5′-CGAACTGGACACACATACAGTG-3′ as the forward primer and 5′-CTGAGGATCTCTGGTTGTGGT-3′ as the reverse primer), ZEB1 (5′-GATGATGAATGCGAGTCAGATGC-3′ as the forward primer and 5′-ACAGCAGTGTCTTGTTGTTGT-3′ as the reverse primer), RAC1 (5′-ATGTCCGTGCAAAGTGGTATC-3′ as the forward primer and 5′-CTCGGATCGCTTCGTCAAACA-3′ as the reverse primer), and TB Green Premix Ex Taq II (TaKaRa Bio). In each sample, all gene expression levels were normalized to GAPDH expression as an internal control.

### Knockdown experiments

PANC-1 cells seeded in a 12-well culture plate at a density of 5700 cells /cm^2^ were treated with siRNA and Lipofectamine RNAiMAX (Invitrogen) according to the manufacturer’s instruction on day 0 and day 3. The siRNA concentrations used were 5 nM on day 0 and 10 nM on day 3. Cells were fixed on day 7 to perform immunostaining and quantification of cell clusters.

siRNA duplexes oligonucleotides were obtained from Integrated DNA Technologies (Coralville, IA, USA). Sequences of siRNA duplexes for KIF3A are as follows: forward, 5′-GCA AGA ACG GUU GGA UAU UGA AGA A-3′; reverse, 5′-UUC UUC AAU AUC CAA GCG UUC UUG CUC-3′. Sequences of siRNA duplexes for IFT88 are as follows: forward, 5′-CAA UCU AUG AUA UCG AGG AAU UGG A-3′; reverse, 5′-UCC AAU UCC UCG AUA UCA UAG AUU GGA-3′. Sequences of siRNA duplexes for negative control are as follows: forward, 5′-CGU UAA UCG CGU AUA AUA CGC GUA T-3′; reverse, 5′-AUA CGC GUA UUA UAC GCG AUU AAC GAC-3′.

### Western blot analyses

Cells were solubilized in 1 × sodium dodecyl sulfate–polyacrylamide gel electrophoresis (SDS–PAGE) sample buffer, heated at 95 ºC for 10 min, and loaded on to an acrylamide gel. Following protein transfer, membranes were blocked with 5% bovine serum albumin (BSA) in Tris-buffered saline containing 0.1% Tween-20 (TBST) for 1 h at room temperature. Primary antibodies diluted in 1% BSA/TBST were added, and the membranes were incubated at 4 ºC overnight. After washing with TBST, the membranes were incubated with horseradish peroxidase-conjugated secondary antibody at room temperature for 1 h. Bands were visualized using ECL Prime (GE Healthcare, Chicago, IL, USA) and the signals were detected with VersaDoc 5000 (Bio-Rad, Hercules, CA, USA).

### Quantification

Quantitative image analyses were performed using ImageJ software. The number of cells with primary cilia was calculated by counting the number of ARL13B- or acetylated α-tubulin-positive primary cilia over DAPI-labeled nuclei per sample; at least 150 cells were counted. The images were taken with an epifluorescence microscope (DMI3000B, Leica; Tokyo, Japan) equipped with a 100 × , N.A. 1.40 objective lens.

Cell clusters were detected and quantified by the following procedure. First, thirty random images were taken with an epifluorescence microscope (DMI3000B, Leica; Tokyo, Japan) equipped with a 10 × , N.A. 0.40 objective lens. Next, cell segmentation was measured using the “threshold” tool of ImageJ. The lower and upper threshold values were set to 60 and 255, respectively. The cutoff values were determined while using ImageJ to determine the values at which cell clusters and non-cell clusters could be distinguished. Finally, an area of 10,000 µm^2^ or more was defined as a cell cluster and the number of cell clusters was measured using the “analyze particles” tool of ImageJ.

### Statistical analysis

The results are presented as the mean ± standard deviation (SD). The statistical significance of the difference between two means was determined using a two-tailed unpaired Student’s *t*-test. Group means were compared using one-way ANOVA for differences. Differences with *p* values of < 0.05 were considered to be statistically significant. All statistical calculations were carried out using JMP statistical software, version 16.0 (SAS Institute, Cary, NC, USA).

## Results

### PANC-1 cells in cell cluster grown from a solitary cell have primary cilia

**Fig. 1:**
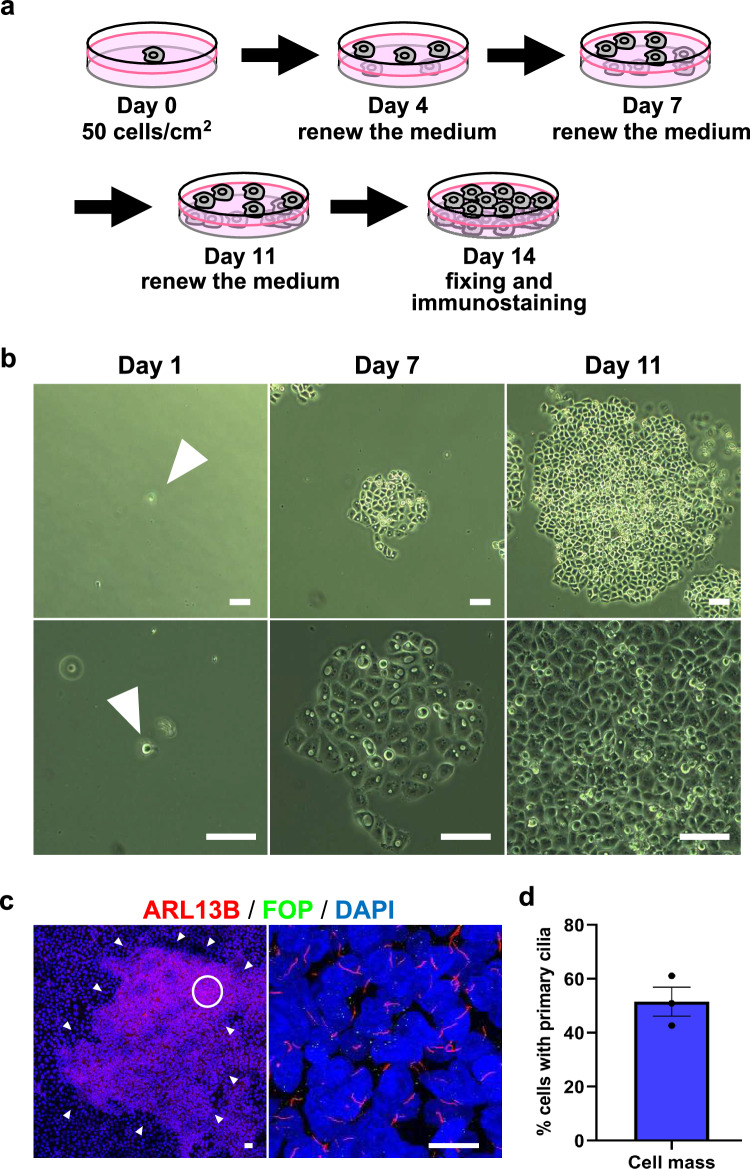
Cells in cell cluster grown from a solitary cell possess primary cilia. **a** The scheme of experiment to grow PANC-1 cells from solitary conditions. The cells were seeded at a density of 50 cells/cm^2^ and cultured for 14 days with the culture medium replaced on days 4, 7, and 11. **b** Representative photographs of a cell cluster grown from a solitary cell. Arrowheads indicate a solitary PANC-1 cell. Scale bar, 100 µm. **c** Primary cilia in cell cluster detected with anti-ARL13B antibodies. Arrowheads indicate the edge of a cell cluster. Cells were immunostained with an anti-ARL13B (red) and anti-FOP (green) antibodies. Nuclei were stained with DAPI (blue). An area surrounded by a circle was magnified. Scale bar, 20 µm. **d** Quantified data of cells with primary cilia in cell cluster. A mean value ± standard deviation from three fields of view is shown

First, to examine whether the growth of PANC-1 cells from solitary condition affects primary cilia formation, we cultured cells under a low density condition. We seeded the cells sparsely at a density of 50 cells/cm^2^ and cultured them for 14 days, changing the medium to a new one on day 4, day 7, and day 11 (Fig. 1a). Under the condition, a solitary PANC-1 cell proliferated to form a cell cluster (Fig. 1b). We visualized primary cilia by staining ARL13B, a primary ciliary marker, and the ciliary base by staining FOP, a centriolar protein, using immunostaining. Immunostaining revealed that a large number of cells in the cell cluster had primary cilia (Fig. 1c). Quantified data showed that about 50% (42.2 to 61.1%) cells possessed primary cilia in the cell cluster (Fig. 1d). These results imply that solitary condition releases potentials of primary cilia formation of PANC-1 cells.

### The initial cell density is an important factor of gaining abilities of primary cilia formation

**Fig. 2:**
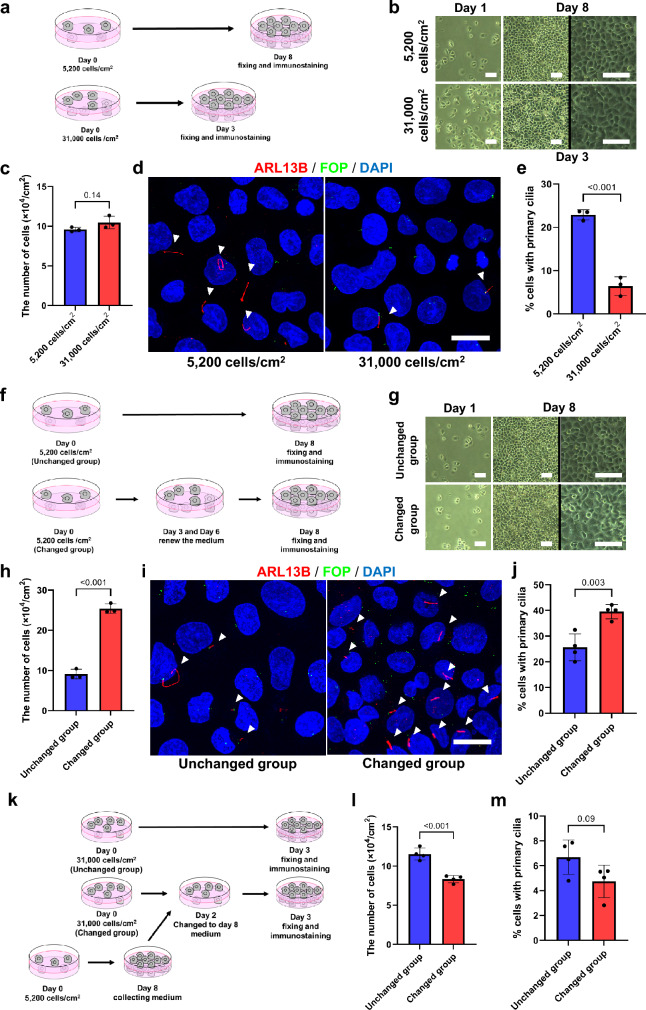
The initial cell density is an important factor for abilities of primary cilia formation. **a** The scheme of experiment to achieve comparable confluency cells from different initial cell density. PANC-1 cells were seeded at a density of 5200 cells/cm^2^ or 31,000 cells/cm^2^ for 8 or 3 days, respectively. **b** Representative photographs showing the cell proliferation and density. Scale bar, 100 µm. **c** Quantified data of cell density in (**b**). An average value ± standard deviation of three independent experiments is shown. **d** Primary cilia in confluent PANC-1 cells detected with anti-ARL13B antibodies. Arrowheads indicate primary cilia. Cells were immunostained with anti-ARL13B (red) and anti-FOP (green) antibodies. Nuclei were stained with DAPI (blue). Scale bar, 20 µm. **e** Quantified data of cells with primary cilia in (**d**). An average value ± standard deviation of three independent experiments is shown. **f** The scheme of experiments for (**g**–**j**). PANC-1 cells seeded at a density of 5200 cells/cm^2^ were cultured for 8 days with the medium unchanged or changed every 3 days. **g** Representative photographs showing the cell proliferation and density. Scale bar, 100 µm. **h** Quantified data of cell density in (**g**). A mean value ± standard deviation of three independent experiments is shown. **i** Primary cilia in the 8-day culture of PANC-1 cells. Cells were immunostained as in (**d**). Arrowheads indicate primary cilia. Scale bar, 20 µm. **j** Quantified data of cells with primary cilia in (**i**). A mean value ± standard deviation of four independent experiments is shown. **k** The scheme of experiments for (**l**, **m**). PANC-1 cells seeded at a density of 31,000 cells/cm^2^ were cultured for 3 days with the medium unchanged or changed to 8-day consumed medium. **l** Comparison of cell density between medium unchanged and changed group. A mean value ± standard deviation of four independent experiments is shown. **m** Comparison of the rate of primary cilia-positive cells. A mean value ± standard deviation of four independent experiments is shown. In **e**, **h**, **j**, **l**, and **m**, *p* < 0.05 was defined as statistically significant, performing the two-tailed unpaired Student’s *t*-test

We next attempted seeking out a key factor that induces primary cilia formation in the cell cluster grown from a solitary cell. We hypothesized that the initial low density, i.e. solitariness, of cells was important for gaining competency of primary cilia formation. To examine it, we made a condition where the final cell density was comparable between two cultures with different initial cell density and culture period (Fig. 1a). To this end, we seeded PANC-1 cells at 5200 cells/cm^2^ or 31,000 cells/cm^2^, and cultured them for 8 days or 3 days, respectively (Fig. 2a and b). The cell density was 9.57 ± 0.29 × 10^4^ cells/cm^3^ in the dish cultured for 8 days from 5200 cells/cm^2^, and 10.46 ± 0.78 × 10^4^ cells/cm^2^ in the dish cultured for 3 days from 31,000 cells/cm^2^ (*p* = 0.14) (Fig. 2c). Cells grown from less cell density possessed primary cilia more frequently; the percentages of primary cilia-possessing cells were 22.9 ± 1.2% in the 8-day culture from 5200 cells/cm^2^ and 6.4 ± 2.2% in the 3-day culture from 31,000 cells/cm^2^ (*p* < 0.001) (Fig. 2d and 2e).

A nutrient starvation has been reported to induce primary cilia formation in several types of cultured cells including PANC-1 cells [32–34]. This raises a possibility that the higher primary cilia formation in culture at 5200 cells/cm^2^ could result from depletion of nutrient during the longer 8-day culture than shorter 3-day culture. To rule out this possibility, we designed two types of experiments. In one experiment, we refilled nutrients by replacing the culture medium with fresh one during the 8-day culture from the cell density of 5200 cells/cm^2^ (Fig. 2f). The number of cells was increased by the medium replacement; 9.10 ± 1.18 × 10^4^ cells/cm^2^ in the group that medium was not replaced versus 25.38 ± 1.21 × 10^4^ cells/cm^2^ in the group that medium was refreshed (*p* < 0.001) (Fig. 2g and h). This indicates that refilled nutrients enhance cell proliferation. Importantly, preventing nutrient depletion failed to suppress primary cilia formation. Rather, the percentages of cells with primary cilia was increased about 50%; 25.6 ± 5.2% in the group that medium was not replaced versus 39.6 ± 2.8% in the group that medium was refreshed (Fig. 2i and j).

In the other experiment, we exposed cells grown from 31,000 cells/cm^2^ to nutrient starvation by using conditioned medium that was taken from 8-day low density culture where cells were seeded at 5200 cells/cm^2^ (Fig. 2k). Under this condition, the number of cells was decreased about 30%; 11.53 ± 0.78 × 10^4^ cells /cm^2^ in the group that medium was not replaced versus 8.33 ± 0.47 × 10^4^ cells/cm^2^ in the group that medium was replaced (*p* < 0.001) (Fig. 2l). Exposing cells to nutrient starvation failed to enhance primary cilia formation; the percentages of cells with primary cilia were 6.7 ± 1.4% in the group that medium was not replaced versus 4.7 ± 1.3% in the group that medium was replaced (*p* = 0.09) (Fig. 2m). These results indicate that the increase in primary cilia formation in the longer culture period from the lower cell density is not a result from nutrient starvation of the medium and imply that PANC-1 cells grown from solitary conditions are able to form primary cilia more readily than those kept in mass culture.

We also attempted to examine the effect of initial cell density on the ability to form primary cilia in another human pancreatic cancer cell line, MIA PaCa-2, which was reported to possess primary cilia [32]. We seeded MIA PaCa-2 cells at 1,700 cells/cm^2^ or 31,000 cells/cm^2^, and cultured them for 5 days or 2 days, respectively (Fig. S1a and S1b). As confluency was approached, MIA PaCa-2 cells began to die and the cell density did not reach confluency (Fig. S1b, right panels). The cell density was 7.57 ± 1.26 × 10^4^ cells/cm^2^ in the dish cultured for 5 days from 1700 cells/cm^2^, and 8.50 ± 0.82 × 10^4^ cells/cm^2^ in the dish cultured for 2 days from 31,000 cells/cm^2^ (*p* = 0.27) (Fig. S1c). As primary cilia of MIA PaCa-2 cells were not positive for ARL13B [32], primary cilia were detected with acetylated α-tubulin used as their marker albeit the length was short as reported [32] (Fig. S1d). The ability of primary cilia formation in MIA PaCa-2 cells was almost comparable between two cultures with different initial cell densities; the percentages of primary cilia-possessing cells were 36.5 ± 12.2% in the 5-day culture from 1700 cells/cm^2^ and 39.0 ± 7.25% in the 2-day culture from 31,000 cells/cm^2^ (*p* = 0.74) (Fig. S1e and S1f). Therefore, the changes of primary cilia formation abilities depending on initial cell density seem to be specific to PANC-1 cells.

### PANC-1 cells passing through limiting dilution possess high ability of primary cilia formation

**Fig. 3:**
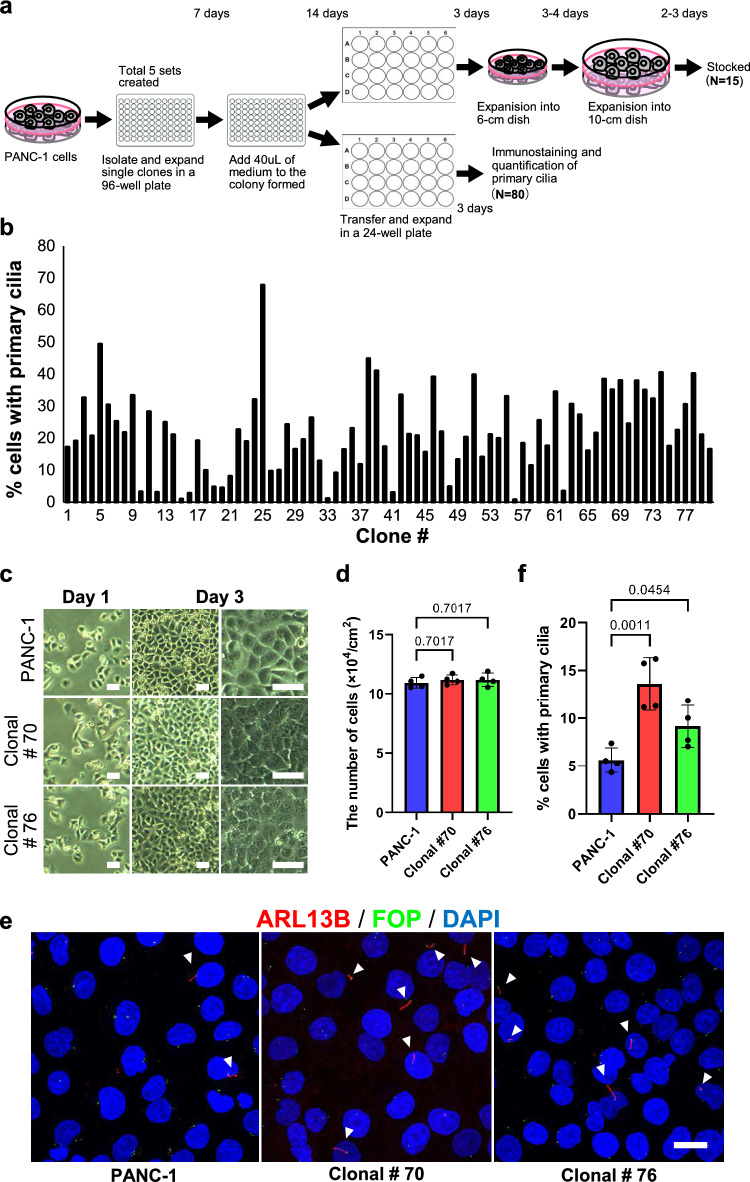
PANC-1 cells passing through limiting dilution possess high ability of primary cilia formation. **a** The scheme of experiment to acquire PANC-1 clones grown from a solitary cell. Clones grown in 96-well plates after limiting dilution were expanded and subjected to experiments. **b** The percentage of primary cilia formation in 80 PANC-1 clones. All clones had abilities to form primary cilia. **c** Representative photographs showing the cell shape and proliferation of parental PANC-1 cells and two clones (#70 and #76). Scale bar, 5 µm. **d** Quantified data of cell density on day 3 in (**c**). A mean value ± standard deviation of four independent experiments is shown.** e** Primary cilia in confluent PANC-1 cells detected with anti-ARL13B antibodies. Arrowheads indicate primary cilia. Cells were immunostained with anti-ARL13B (red) and anti-FOP (green) antibodies. Nuclei were stained with DAPI (blue). Scale bar, 20 µm. **f** Quantified data of cells with primary cilia in (**e**). A mean value ± standard deviation of four independent experiments is shown. In **d** and **f**, *p* < 0.05 was defined as statistically significant, performing the one-way ANOVA

To test whether the solitariness of PANC-1 cells is the defining factor of gaining ability of primary cilia formation, we subjected PANC-1 cells to a limiting dilution method (Fig. 3a). It is noteworthy that all clones possess the ability of primary cilia formation; 80/80 clones protruded primary cilia (Fig. 3b). This is highly contrasting to the maximal percentage of PANC-1 cells positive for primary cilia in 8-day culture from low density conditions, 40% (Fig. 2j). The results suggest that PANC-1 cells that pass through a solitary condition acquire ability of primary cilia formation.

We further examined whether PANC-1 cell clones grown from solitary cells exhibit higher primary cilia formation. To this end, we cultured five randomly selected clones and parental PANC-1 cells with the same density and condition as in Fig. 2a. Among the five clones, four clones proliferated at levels comparable to parental PANC-1 cells for 3 days after seeded at 31,000 cells/cm^2^; parental PANC-1 10.9 ± 0.4 × 10^4^ cells/cm^2^, clone #70 11.2 ± 0.4 × 10^4^ cells/cm^2^, clone #76 11.2 ± 0.6 × 10^4^ cells/cm^2^, clone #2 13.0 ± 1.8 × 10^4^ cells/cm^2^, and clone #16 13.3 ± 1.4 × 10^4^ cells/cm^2^ (Fig. 3c, d, S2a and S2b). Only clone #24 showed a slight decrease in cell proliferation and failed to reach full confluency at 3 days (10.3 ± 0.5 × 10^4^ cells/cm^2^, *p* = 0.0046, S2a and S2b). Under the same culture period, five clones showed higher rate of primary cilia formation than parental PANC-1 cells (Fig. 3e, f, Fig. S2c and S2d; parental PANC-1, 5.6 ± 1.3%; clone #70, 13.6 ± 2.7%; clone #76, 9.2 ± 2.2%; clone #2, 14.1 ± 0.3%; clone #16, 21.7 ± 2.7%; clone #24, 10.7 ± 4.3%). Clone #24 also showed an increasing trend of primary cilia formation (10.7 ± 4.3%; ~ 1.9-fold higher than parental PANC-1) albeit it was slightly over the statistical significance criterion (p = 0.0647 > 0.05), as the clone had a less cell proliferation than other clones (Fig. S2b and S2c). These results indicate that PANC-1 cells that passed through solitary conditions acquire higher ability of primary cilia formation.

### The mRNA expression levels of EMT markers change in clonal PANC-1 cells

**Fig. 4:**
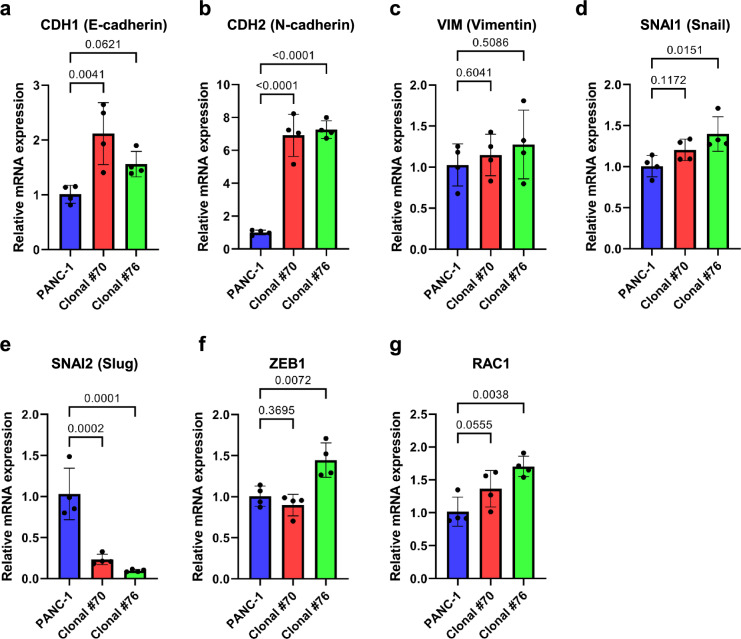
Clonal PANC-1 cells show a significant increase in N-cadherin mRNA expression and a decrease in Slug mRNA expression compared to the parental PANC-1 cells. Quantified data using qPCR to measure mRNA expression of the EMT markers; **a** E-cadherin (CDH1), **b** N-cadherin (CDH2), **c** Vimentin (VIM), **d** Snail (SNAI1), **e** Slug (SNAI2), **f** ZEB1, **g** RAC1. An average value ± standard deviation of four independent experiments is shown. P-values were calculated using the one-way ANOVA

Clonal PANC-1 cells showed a cell shape similar to that of mesenchymal cells (Fig. 3c). We thus investigated a possibility of epithelial-to-mesenchymal transition (EMT) occurring in clonal PANC-1 cells by examining mRNA expression of EMT markers by means of qPCR (Fig. 4). Total RNA was collected from the parental PANC-1 cells and clonal PANC-1 cells (#70 and #76) at 3 days after seeding, and the mRNA expression of the following EMT markers was examined by real-time qPCR; E-cadherin (gene symbol: CDH1), N-cadherin (gene symbol: CDH2), Vimentin (gene symbol: VIM), Snail (gene symbol: SNAI1), Slug (gene symbol: SNAI2), ZEB1 and RAC1. N-cadherin mRNA expression was markedly increased in the both clonal PANC-1 cells compared to the parental PANC-1 cells (Fig. 4b), while Slug was markedly decreased in the both clonal PANC-1 cells compared to the parental PANC-1 cells (Fig. 4e). These results suggest that PANC-1 cells that pass through a solitary condition acquire properties of mesenchymal cells by EMT.

### Cell cluster formation of PANC-1 cells depends on primary cilia formation

**Fig. 5:**
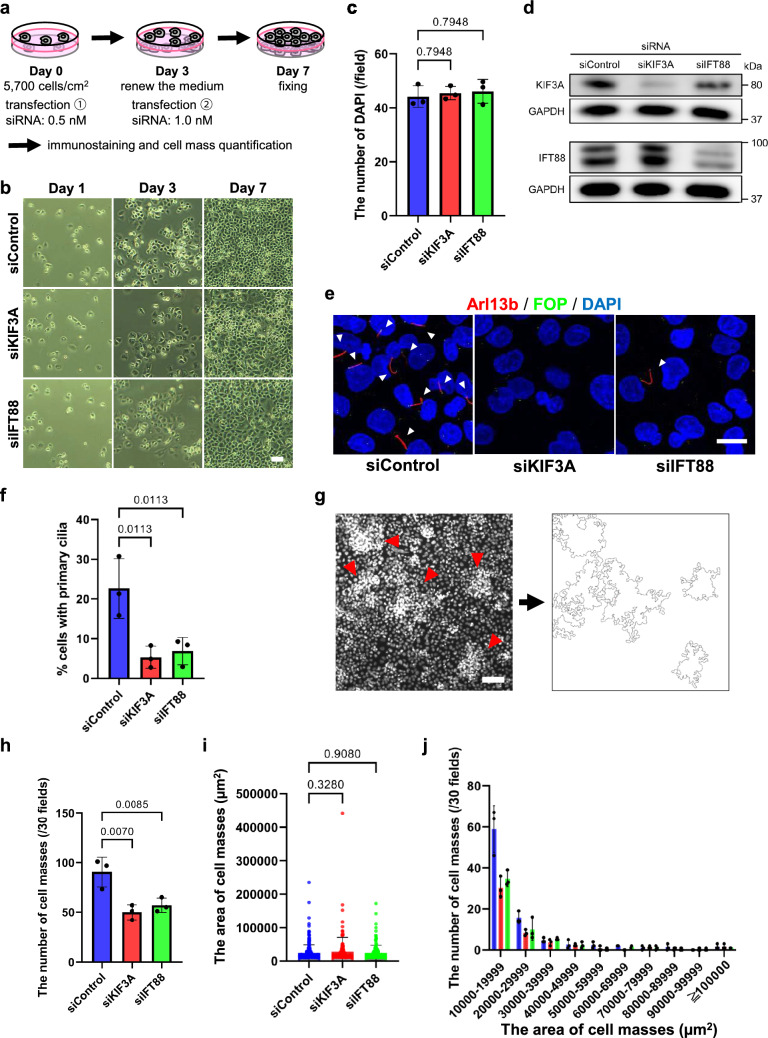
Cell cluster formation of PANC-1 cells depends on primary cilia formation. **a** Experimental scheme of knockdown. Cells were treated twice with siRNA on day 0 and day 3. **b** Representative photographs showing the cell shape and proliferation of siRNA-treated PANC-1 cells. Scale bar, 100 µm. **c** Quantified data of the number of cells per field, in which DAPI-stained nuclei were counted. **d** Western blot analyses verifying knockdown of KIF3A and IFT88. GAPDH was used as a loading control. **e** Representative photographs verifying effects of knockdown of KIF3A and IFT88 on primary cilia formation. Cells were immunostained with an anti-ARL13B (red) and anti-FOP (green) antibodies. Nuclei were stained with DAPI (blue). Arrowheads indicate primary cilia. Scale bar, 20 µm. **f** Quantified data of cells with primary cilia in (**e**). **g** An example of cell cluster quantification using the “threshold” and “analyze particles” tool of ImageJ. **h** Quantified data of cell cluster formation. A mean value ± standard deviation of three independent experiments is shown. **i** Quantified data of the size of cell clusters formed. A mean value ± standard deviation of three independent experiments and the plot for each cluster size are shown. **j** Quantified data of the number of cell clusters in categorized cluster size. A mean value ± standard deviation of three independent experiments is shown. *p* < 0.05 was defined as statistically significant, performing the one-way ANOVA

We finally examined whether cell cluster formation was dependent on primary cilia formation in PANC-1 cells. To this end, we tandemly knocked down KIF3A or IFT88, which were indispensable proteins for primary cilia formation, by using siRNA duplex oligonucleotides (Fig. 5a). PANC-1 cells proliferated under the tandem treatment with siRNA duplex oligonucleotides (Fig. 5b). PANC-1 cells that were subjected to knockdown of KIF3A or IFT88 and parental PANC-1 cells exhibited no significant difference in cell proliferation ability (Fig. 5c; the number of DAPI per field; siControl, 44.2 ± 4.0; siKIF3A, 45.5 ± 2.5; siIFT88, 46.1 ± 4.4). The knockdown efficiency of KIF3A and IFT88 by the tandem siRNA treatment was verified with western blot analyses; siRNA against KIF3A and IFT88 specifically decreased the protein amount of each a target protein, respectively (Fig. 5d). The effects of knockdown on primary cilia formation were verified with immunocytochemistry by detecting primary cilia with anti-ARL13B antibodies (Fig. 5e). Knockdown of KIF3A or IFT88 resulted in remarkable decrease in the number of primary cilia formation in PANC-1 cells (Fig. 5e and f; siControl, 22.6 ± 7.5%; siKIF3A, 5.3 ± 2.8%; siIFT88, 6.9 ± 3.4%).

We compared the number of cell clusters in cells with KIF3A or IFT88 knocked down to that in control cells (Fig. 5g). The number of cell clusters was decreased in PANC-1 cells, in which KIF3A or IFT88 was knocked down, compared to the control PANC-1 cells treated with control siRNA (Fig. 5h; control 90.3 ± 15.1 cell clusters/30 fields, siKIF3A 50.0 ± 4.4 cell clusters/30 fields, siIFT88 57.0 ± 7.2 cell clusters/30 fields). The size of cell clusters, once the clusters were formed, exhibited no significant difference between the parental or knockdown PANC-1 cells (Fig. 5i; the average of cluster size; siControl, 24,180 µm^2^; siKIF3A, 28,225 µm^2^; siIFT88, 24,513 µm^2^). Both the clusters in parental and knockdown PANC-1 cells were mostly in the range of 1000 to 1999 µm^2^ (Fig. 5j). These results suggest that cell cluster formation is dependent on primary cilia formation in PANC-1 cells.

## Discussion

In this work, we demonstrated that about half of PANC-1 cells in cell clusters possessed primary cilia even in the presence of serum (Fig. 1). The rate of primary cilia-positive PANC-1 cells in cell clusters is comparable of or more than previously reported maximal rates under serum-deprived conditions [27]. Interestingly, the high primary cilia-positive rate is achieved by culturing cells from lower cell density to form cell clusters (Fig. 2). Only a high cell density does not induce ciliogensis (Fig. 2). These suggest that growth from solitary cells, rather than final cell density, decides the ability of the primary ciliogenesis. A possibility is that this occurs through the selection of cells with high potential of primary ciliogenesis. The other possibility is that some genetic changes occur during the dozens of proliferation from a solitary cell that could increases the ability of primary ciliogenesis. If the former is true, the mixture of cells that have different competencies of primary ciliogenesis could underlie the heterogeneity of PDACs.

We discuss possible pathophysiological roles for the high rate of primary ciliogenesis in cell clusters grown from solitary cells. It would underlie the strong drug resistance of PDAC. PANC-1 cells are reported to acquire resistance against cisplatin dependently on primary ciliogenesis [35]. Conversely, blockade of primary ciliogenesis seems to attenuate the resistance of PANC-1 cells to cisplatin [35]. The high rate of primary ciliogenesis in cell clusters grown from solitary cells could also contribute to acquisition of cancer stemness, as cancer stem cells are thought to have chemotherapy resistance [36–38]. In neoplastic breast tissue cells, primary ciliogensis seems to be required for keeping stemness of the cells [39]. In our experiments especially using PANC-1 clones that passed limiting dilution, similar genetic or transcriptional changes could occur to make cells have stem-like characteristics along with the increase in ability of primary ciliogenesis. Further studies with the PANC-1 clones that have high potencies of primary ciliogenesis are needed.

In MIA PaCa-2 cells, the initial cell density has no effect on the ability to form primary cilia (Fig. S1). While both PANC-1 cells and MIA PaCa-2 cells are derived from human PDAC, it has been reported that some of their properties differ [40, 41]. It has been reported that PANC-1 cells have a higher sphere-forming ability than MIA PaCa-2 cells [42]. In this study, MIA PaCa-2 cells began to die just before reaching confluency (Fig. S1b). Similar to the results of this study, MIA PaCa-2 cells have been reported to form ARL13B-netative and acetylated-α-tubulin-positive markedly short primary cilia [32]. It is likely that the increase in the ability of primary cilia formation dependent on sparse cell density could be a phenomenon or property specific to a type of PDAC cells, which could be involved in the heterogeneity of PDACs,. and further studies are needed to clarify the cause of this difference.

PANC-1 cells grown from a solitary cell appear to exhibit mesenchyme-like cell morphology (Fig. 3c). In clonal PANC-1 cells, N-cadherin mRNA expression increases markedly, while Slug mRNA decreases markedly (Fig. 4). N-cadherin is one of the mesenchymal markers that increases during epithelial-mesenchymal transition (EMT) [46]. Slug is also generally known to be elevated in mesenchymal cells and to contribute to cancer cell invasion [43]. Although it is rarely reported in PDAC, Slug is known to promote EMT and to be highly expressed in human PDAC [44, 45]. Meanwhile, it has been reported that Slug is more highly expressed in basal epithelial cancer cells than in luminal epithelial cancer cells, and less highly expressed in mesenchymal cancer cells than in basal epithelial cancer cells [46, 47]. It is possible that by solitary culturing the parental PANC-1 cells, which are basal epithelial cancer cells, become the mesenchymal cancer cells through EMT. EMT is a process by which epithelial cells transform into mesenchyme-like cells by losing their cell polarity and the function of cell adhesion with surrounding cells and gaining migratory and invasive capacities. Some studies about cancers other than PDACs report that EMT could be involved in primary ciliogenesis. In breast cancer, key EMT programs induce primary ciliogenesis and its downstream Hedgehog signaling [48]. EMT transcription factors also enhance primary ciliogenesis by inducing expression of positive regulators for ciliogenesis, which in turn promotes tumorigenesis [49]. A subgroup of clear cell renal cell carcinomas also have primary cilia along with EMT characteristics, showing tumor aggressiveness and poor prognosis [50]. These reports suggest that transcription factors and intracellular signals that induce EMT promote the primary ciliogenesis. Given the mesenchyme-like morphology of PANC-1 cells grown from solitary cells in our experiments, an idea could be proposed that cells possessing potencies of EMT survive through solitary conditions with the ability of primary ciliogenesis kept or acquired in PDACs.

Finally, knockdown of KIF3A and IFT88 by siRNA reduced primary ciliogenesis and cluster formation in solitary cell-derived PANC-1 cells (Fig. 5). This appears contradictory to commonly accepted scenario for roles of primary cilia in cell proliferation. Primary cilia are formed during a quiescent phase of the cell cycle, i.e. G0 phase, and in general understandings cancer cells that do not assemble primary cilia grow faster and are more malignant [51–54]. However, recent works provide arguable findings that primary cilia function as both tumor suppressors and tumor promoters, depending on various conditions such as cancer types and environment [55]. Both tumor suppressive and promotive roles are also reported in PANC-1 cells. Classically, suppression of primary ciliogenesis appears to promote PANC-1 cell proliferation [27, 28]. In contrast, a recent work reports that restoration of primary cilia enhances PANC-1 cell proliferation [29]. Our finding is consistent with the recent work. A clinical work also supports our finding. Lymph node metastasis positively correlates with the presence of primary cilia in PDAC patients [30]. While different outcomes are observed in different environments that cause the formation or non-formation of primary cilia in PDAC, at least, the results of this study suggest that cell proliferation and cell cluster formation are promoted by the increased ability of primary ciliogenesis in PANC-1 cells grown from solitary cells. The findings of this study could explain a mechanistic insight on the high metastatic potential and poor prognosis of PDAC with primary cilia.

In conclusion, we herein presented that a type of pancreatic cancer cells grown from solitary conditions form cell clusters with increased primary ciliogenesis and that the cell cluster formation was primary ciliogenesis-dependent. Given that the very high metastatic potential of a type of PDAC is achieved due to the high clustering potential of PDAC when it becomes a CTC and becomes monocytic at the metastatic site [56], measurement of CTCs is proposed to be useful to assess the risk of recurrence of PDAC [57]. Establishment of a method to detect malignant PDAC-derived CTCs is expected for early detection and treatment of pancreatic cancer. Primary cilia could be used a marker for PDAC-derived CTCs with high metastatic potential. Further research is needed to understand mechanisms that underlie the increase in primary ciliogenesis through growth from solitary cells and primary ciliogenesis-dependent tumor cluster formation.

## Supplementary Information

Below is the link to the electronic supplementary material.

Supplementary file 1 (PDF 912 KB) Supplemental Figure 1 The initial cell density does not affect the primary cilia formation ability in MIA PaCa-2 cells. a The scheme of experiments to achieve comparable sub-confluency cells from different initial cell density. MIA PaCa-2 cells were seeded at a density of 1,700 cells/cm2 or 31,000 cells/cm2, and cultured for 5 or 2 days, respectively. b Representative photographs showing the cell proliferation and density. Scale bar, 100 µm. c Quantified data of cell density at 5 days (1,700 cells/cm2) or 2 days (31,000 cells/cm2) in b. An average value ± standard deviation of four independent experiments is shown. d Primary cilia in sub-confluent MIA PaCa-2 cells detected with anti-acetylated α-tubulin antibodies. Representative images of primary cilia-positive cells (upper panels) and primary cilia-negative cells (lower panels). White arrowheads indicate centrosome with primary cilia. Yellow arrowheads indicate centrosome without primary cilia. Cells were immunostained with anti-acetylated α-tubulin (red) and γ-tubulin (green) antibodies. Nuclei were stained with DAPI (blue). Scale bar, 2 µm. e Primary cilia were detected in MIA PaCa-2 cells at 5 days after seeding (1,700 cells/cm2) or at 2 days after seeding (31,000 cells/cm2). Arrowheads indicate primary cilia. Cells were immunostained with anti-acetylated α-tubulin (red) and γ-tubulin (green) antibodies. Nuclei were stained with DAPI (blue). Scale bar, 20 µm. f Quantified data of cells with primary cilia in e. An average value ± standard deviation of four independent experiments is shown. P-values were calculated using a two-tailed unpaired Student’s t-test. Supplemental Figure 2 Other randomly selected PANC-1 clones also exhibit high ability of primary cilia formation. a Representative photographs showing the cell shape and proliferation of parental PANC-1 cells and three clones (#2, #16 and #24). Scale bar, 5 µm. b Quantified data of cell density on day 3 in a. A mean value ± standard deviation of four independent experiments is shown. c Primary cilia in confluent PANC-1 cells detected with anti-ARL13B antibodies. Arrowheads indicate primary cilia. Cells were immunostained with anti-ARL13B (red) and anti-FOP (green) antibodies. Nuclei were stained with DAPI (blue). Scale bar, 20 µm. d Quantified data of cells with primary cilia in c. A mean value ± standard deviation of four independent experiments is shown. In b and d, p<0.05 was defined as statistically significant, performing the one-way ANOVA.
