# Supplementary figures and images for: A type of pancreatic cancer cells form cell clusters from a solitary condition in a primary ciliogenesis-dependent manner
Source: Med Mol Morphol. 2025 Mar 12;58(3):213–26. doi: 10.1007/s00795-025-00428-0 (PMC12378286; doi:10.1007/s00795-025-00428-0)

# Supplemental Figure 1

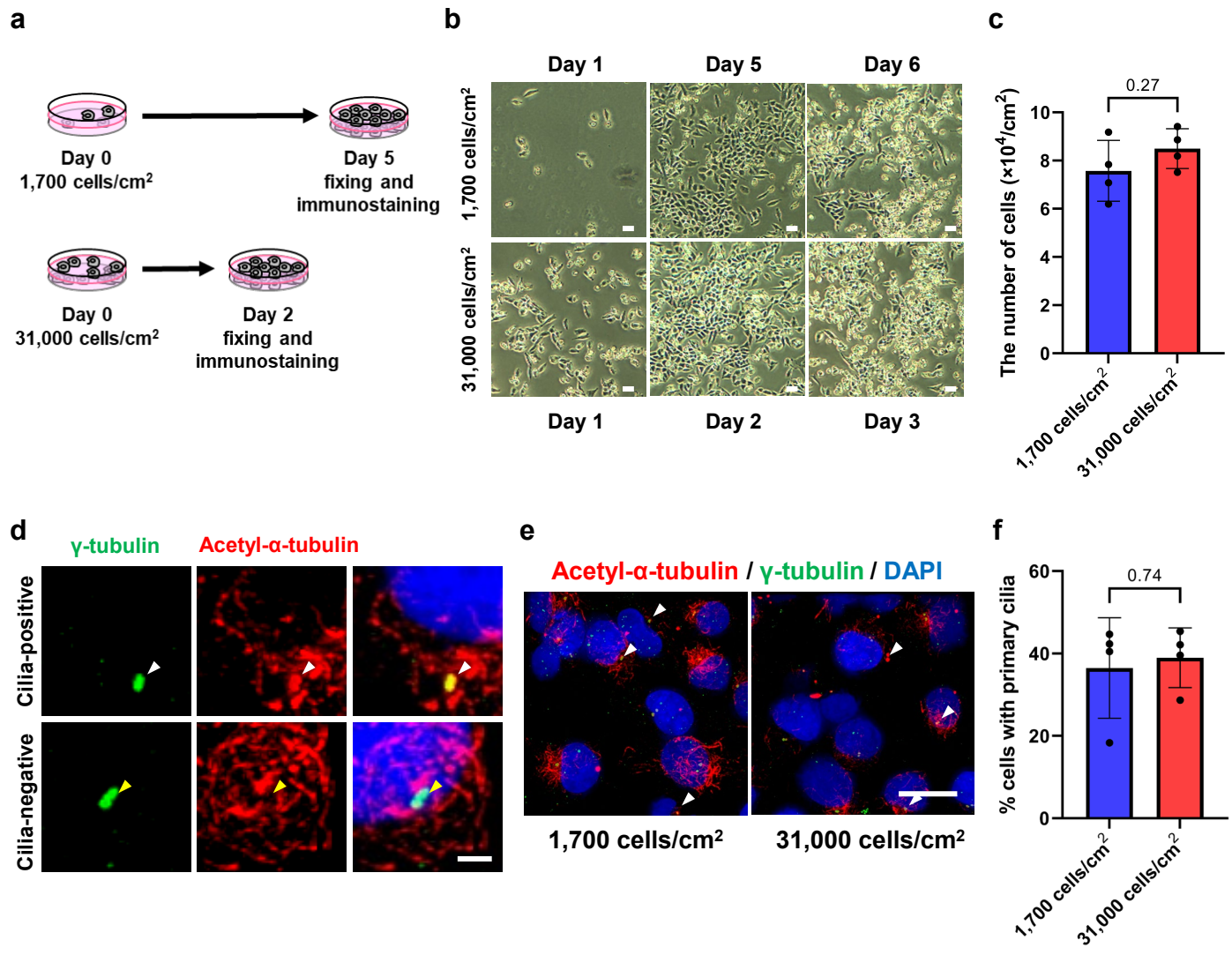

# Supplemental Figure 2

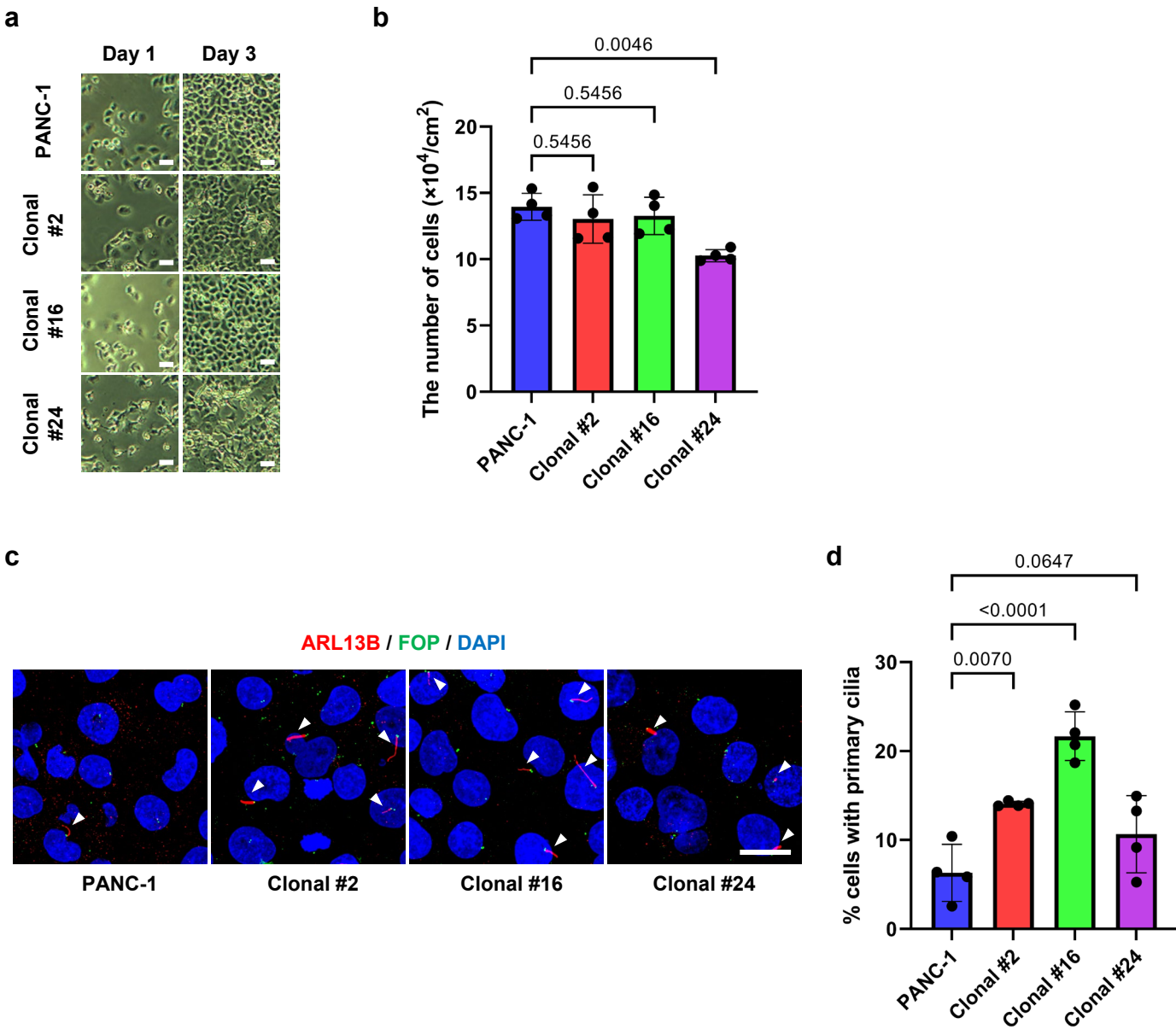

Supplement: Supplementary file 1 — (PDF 912 KB) Supplemental Figure 1 The initial cell density does not affect the primary cilia formation ability in MIA PaCa-2 cells. a The scheme of experiments to achieve comparable sub-confluency cells from different initial cell density. MIA PaCa-2 cells were seeded at a density of 1,700 cells/cm2 or 31,000 cells/cm2, and cultured for 5 or 2 days, respectively. b Representative photographs showing the cell proliferation and density. Scale bar, 100 µm. c Quantified data of cell density at 5 days (1,700 cells/cm2) or 2 days (31,000 cells/cm2) in b. An average value ± standard deviation of four independent experiments is shown. d Primary cilia in sub-confluent MIA PaCa-2 cells detected with anti-acetylated α-tubulin antibodies. Representative images of primary cilia-positive cells (upper panels) and primary cilia-negative cells (lower panels). White arrowheads indicate centrosome with primary cilia. Yellow arrowheads indicate centrosome without primary cilia. Cells were immunostained with anti-acetylated α-tubulin (red) and γ-tubulin (green) antibodies. Nuclei were stained with DAPI (blue). Scale bar, 2 µm. e Primary cilia were detected in MIA PaCa-2 cells at 5 days after seeding (1,700 cells/cm2) or at 2 days after seeding (31,000 cells/cm2). Arrowheads indicate primary cilia. Cells were immunostained with anti-acetylated α-tubulin (red) and γ-tubulin (green) antibodies. Nuclei were stained with DAPI (blue). Scale bar, 20 µm. f Quantified data of cells with primary cilia in e. An average value ± standard deviation of four independent experiments is shown. P-values were calculated using a two-tailed unpaired Student’s t-test. Supplemental Figure 2 Other randomly selected PANC-1 clones also exhibit high ability of primary cilia formation. a Representative photographs showing the cell shape and proliferation of parental PANC-1 cells and three clones (#2, #16 and #24). Scale bar, 5 µm. b Quantified data of cell density on day 3 in a. A mean value ± [file 795_2025_428_MOESM1_ESM.pdf]
